# Targeted environmental enrichment is more effective than bipedal treadmill training after thoracic spinal cord injury

**DOI:** 10.1093/braincomms/fcaf385

**Published:** 2025-10-06

**Authors:** Jarred M Griffin, Till Bockemühl, Blanca Randel, Stefanie Gröschl, Panagiotis Papaioannou, Ansgar Büschges, Frank Bradke

**Affiliations:** Cure Programme, Faculty of Medical and Health Sciences, The University of Auckland, Auckland 1023, New Zealand; School of Pharmacy, Faculty of Medical and Health Sciences, The University of Auckland, Auckland 1023, New Zealand; Laboratory for Axonal Growth and Regeneration, German Center for Neurodegenerative Diseases (DZNE), Bonn 53127, Germany; Department of Animal Physiology, Institute of Zoology, University of Cologne, Cologne 50674, Germany; Laboratory for Axonal Growth and Regeneration, German Center for Neurodegenerative Diseases (DZNE), Bonn 53127, Germany; Laboratory for Axonal Growth and Regeneration, German Center for Neurodegenerative Diseases (DZNE), Bonn 53127, Germany; Cure Programme, Faculty of Medical and Health Sciences, The University of Auckland, Auckland 1023, New Zealand; School of Pharmacy, Faculty of Medical and Health Sciences, The University of Auckland, Auckland 1023, New Zealand; Department of Animal Physiology, Institute of Zoology, University of Cologne, Cologne 50674, Germany; Laboratory for Axonal Growth and Regeneration, German Center for Neurodegenerative Diseases (DZNE), Bonn 53127, Germany

**Keywords:** spinal cord injury, epothilone, rehabilitation, targeted environmental enrichment, bipedal treadmill training

## Abstract

Rehabilitation is widely recognized as an essential component in evaluating the effectiveness of a therapeutic intervention in animal models of translational spinal cord injury research. Ideally, the rehabilitation method used should be optimized for the specific injury model and severity to adequately compare the efficacy of the investigational therapy. For studies utilizing thoracic spinal cord injuries, rehabilitation primarily involves bipedal or quadrupedal treadmill training aimed at restoring hindlimb function. Previously, we reported modest improvements to hindlimb motor recovery following forced bipedal treadmill training after moderate 175 kilodyne thoracic spinal cord contusion injury, which were further enhanced when combined with the microtubule-stabilizing drug epothilone B. However, during this and other studies we have noted that B-TMT presents several potential limitations, including brief periods of active rehabilitation for each animal, variability between animal handlers, animal stress, and the focus on a single movement pattern. To overcome these limitations, we investigated the effects of an environmental enrichment-based rehabilitation that differs from a general environment enrichment strategy by including task-specific training elements, something we have termed ‘targeted environmental enrichment’. This method provides longer periods of active rehabilitation, operates independently of the animal handler, thereby involves less animal handling, which minimizes stress, and encourages a wide range of movements. Our findings indicate that targeted environmental enrichment outperforms bipedal training across several behavioural measures which produced a ceiling effect that epothilone B could not overcome. Therefore, these results suggest that targeted environmental enrichment may be a more effective rehabilitation approach than B-TMT and should be considered for application in models of severe spinal cord injury.

## Teaser

Griffin *et al*. found targeted environmental enrichment proved more effective than B-TMT in thoracic spinal cord injury models. It enabled more consistent and diverse motor engagement, promoted naturalistic behaviours, and supported broader functional recovery. This approach may better support recovery in severe spinal cord injury models.

## Graphical Abstract

**Graphical Abstract:**
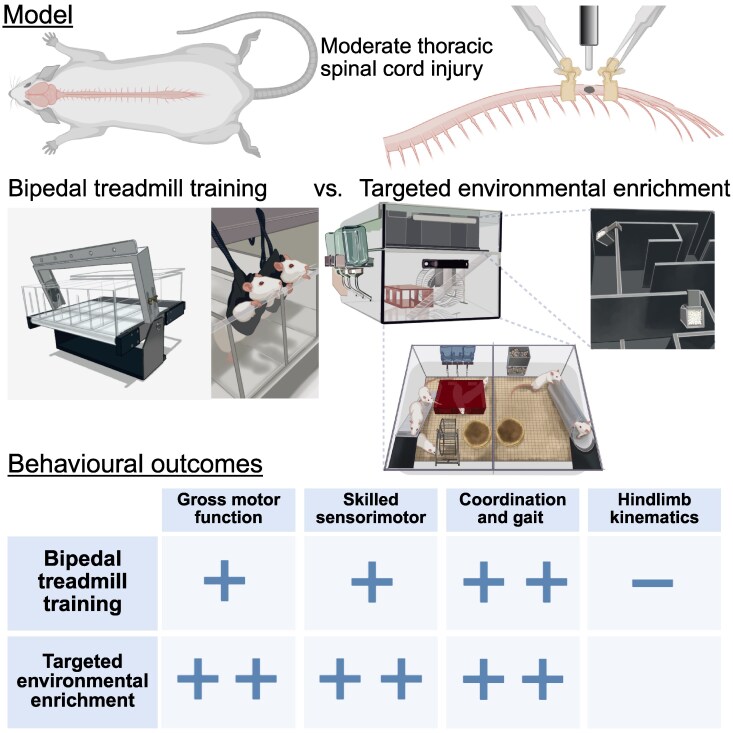

## Introduction

Rehabilitation is almost invariably provided to people who have suffered a spinal cord injury (SCI).^1,2^ Therefore, when it comes to translational studies in animal models, it appears crucial to combine rehabilitation with the investigated therapeutic agent.^3-5^ Various rehabilitation approaches for rodent models have emerged over the years, differing in the training type, task-specificity, onset, duration and intensity.^6^ However, the majority of rehabilitation approaches after thoracic SCIs are through bipedal or quadrupedal treadmill training in efforts to be most engaging with the paralyzed hindlimbs.^3,6^ In line with this, we previously reported that bipedal treadmill training (B-TMT) can additively improve functional recovery after thoracic SCI, either alone or in combination with epothilone B (epoB) treatment, a microtubule stabilizing drug inducing axon regeneration and reducing fibrotic scarring leading to functional recovery after SCI,^7^ or with an extracellular matrix-degrading gene therapy.^8,9^ During these studies, we reflected on several limitations of this rehabilitation approach: the conceivably minimal duration in which the animals can be exposed to the training, the low reproducibility between handlers, the increased stress level in animals due to the forced task and, lastly, the lack of exposure to a broader range of rehabilitative movements. To address these concerns, we asked whether an environmental enrichment-based approach which includes task-specific training elements could be as effective as B-TMT, hereby named targeted environmental enrichment (T-EE). In this process, we established a standardizable, commercially available T-EE paradigm for the field of motor rehabilitation. This difference to a ‘general environmental enrichment’ (EE) is that T-EE includes task-specific training elements. In comparison to what we consider ‘general EE’, which typically solely includes social housing and nesting materials, T-EE is designed to actively promote functional recovery through deliberate inclusion of rehabilitative tasks. T-EE is characterized by extended periods of active rehabilitation, minimal reliance on the animal handler, reduced animal handling to prevent stress, and most importantly, promotion of diverse movement patterns directed at restoring lost function. Through an array of behavioural measures, we showed that T-EE is more effective than B-TMT. In fact, T-EE produced a ceiling effect in this moderate severity thoracic spinal contusion injury. Our findings suggest that enrichment-based rehabilitation may be a more effective approach and should be considered for use in translational models of severe SCI.

## Materials and methods

Extended methods can be found within the Supplementary material.

### Animals, surgical procedures and epothilone treatment

Adult, female Sprague Dawley rats (∼250 g) were housed in a reverse light cycle. Standard housing within our facility consists of Techniplast 1500 U cages (1500 cm2), with two animals per cage and the enrichment object of one wooden block, nesting material and one shelter object. Animals were administered antibiotics (Enrofloxacin, 5 mg/kg, subcutaneously; s.c.) and analgesics (Meloxicam, 1 mg/kg; s.c.) and then anaesthetized with isoflurane. A laminectomy and a moderate thoracic level 10 (T10) 175 kdyn contusion injury was performed. Animals were administered antibiotics (Enrofloxacin 5 mg/kg, s.c.) and analgesics (Meloxicam 1 mg/kg, Buprenorphine 0.05 mg/kg, s.c.) for 3 days following the surgery. Animals were randomly allocated to intervention groups and received blinded s.c. injections of epoB (0.75 mg/kg), or vehicle [50% dimethyl sulfoxide (DMSO)] on days 1 and 15 post-injury.

### Bipedal treadmill rehabilitation

A custom-adapted five-lane rodent treadmill was used whereby animals were supported upright in harnesses and positioned so that their hind paws touched the surface of the treadmill, as previously described.^8,9^ Training started one week prior to the injury and involved a guided bipedal stepping for 30 min per day. Three weeks post-SCI, rats completed B-TMT at a speed of 20–30 cm/s for 20 min a day, 5 days a week, for 7 weeks in total. This totalled to 400 meters of running on the hindlimbs per day, on average. Immediately following the B-TMT, the rats underwent 20 min of quadrupedal exercise training at a speed of 30–40 cm/s. To illustrate the training process, we have added two Supplementary Videos: one from the pre-injury training phase and another from the post-injury training phase (Supplementary Videos 1 and 2). These highlight both the method used to train the animals to walk bipedally and their ability to perform B-TMT at speeds ranging from 20 to 35 cm/s.

### Targeted environmental enrichment rehabilitation

The Marlau environmental enrichment cage (ViewPoint; length: 800 mm × width: 600 mm × height: 510 mm) served as the basis of our T-EE. This cage consisted of a two-story design with a ground floor consisting of two compartments separated by a one-way door: one contains food pellets and the other water bottles. A weekly-rearranged maze was placed on the upper floor ensuring all animals have equal access to the distinctive features of enrichment, and cognitive stimulation is maintained. Modifications were made to cover the floor with a raised grid to encourage intentional placement of the hindlimbs and grasping of the paws; an irregular rung running wheel was added; balance boards were placed between the partition doors; reward baskets loaded with sugar pellets were hung in the maze and changed position every day to encourage standing and exploratory behaviour. Fourteen rats were placed in the cage together. The animals were exposed to the cage for three days, 2 months prior to the start of the study to avoid a potential enrichment-induced predisposition to improvements.^10^ Each week, recordings of the complete 3 h were made from three viewpoints of the cage, from which the activity of the animals was assessed (Supplementary Videos 3–6).

### Activity analysis

For quantitative analysis of the recordings from the T-EE cage, tracking of individual animals within the cage was conducted at weeks 3, 6, and 9 post-injuries. From each three-hour-long weekly recording, three 5-min-long segments were selected for analysis: 5–10 min, 90–95 min, and 150–155 min. Distance measurements were conducted using Fiji and the MTrackJ plugin. Videos were calibrated to distance and MTrackJ was used to manually track each rat’s *x*/*y* coordinates in mm at every frame of the recording, with the time interval between frames set at 0.2 s. The real distances between successive coordinates were calculated and summed to determine the total distance travelled.

### BBB and horizontal ladder test

The Basso, Beattie and Bresnahan (BBB) locomotor rating scale was conducted in a circular open field for 5 min as previously described.^11^ For the horizontal ladder test, a 100 cm-long ladder with irregularly spaced rungs was used. Each animal completed three runs of the ladder while being recorded using a camera (GoPro Hero5). Steps were considered an error if the paw slipped from the rung, with both partial and full slips included. The average number of errors was recorded over the three runs and expressed as a percentage of the total steps.

### CatWalk gait analysis

Only runs with a maximum run variation of < 60% and completion within 5 s were included, and four successful runs per animal were recorded. Parameters that displayed a fold change of 15% between uninjured and injured animals with a *P* < 0.01 were included in the study (Supplementary Fig. 1, Supplementary Tables 1 and 2). This produced a total of 37 parameters, largely consistent with previous reports.^12^ This data set was centred and each column normalized to a standard deviation of 1 before conducting principal component analysis (PCA). We extracted the explained variance for each principal component (PC) and the coefficients for the first three PCs. Following this, we processed the PCA dataset by collapsing columns with similar annotations to the means and performed heatmap hierarchical clustering to identify correlations between groups.

### Hindlimb kinematic analysis

Markings were made on the iliac crest, hip, ankle, knee and toe joint, and videos of overground walking were recorded at a frame rate of 193 frames/s. The markers were then tracked in the videos using TSE Motion V9.2.2. Resulting *x*/*y* coordinates were used to calculate joint angle time courses. To average all steps in a particular leg, we calculated the movement speed of the foot markers as a proxy for the swing onset of individual steps. Whenever the speed of the foot marker exceeded an empirically determined threshold, we defined this as the onset of a swing phase, i.e. lift-off. Complete step cycles were defined as the movement from one lift-off event to the next. After normalizing each individual step cycle to 100-time units, the joint angle time courses associated with these step cycles were averaged per body sides. We further calculated the additional kinematic measures at swing onset, mid-stance, and touchdown: Iliac crest height, joint angles, stride length, angular and postural variability.

### Statistical analyses

This study partially includes data reanalysed from our previous publication.^9^ This was necessary to enable direct comparisons between the two types of rehabilitation. Exclusion criteria were set prior to the initiation of each cohort within this study (see Supplementary). Unless otherwise stated, all numerical values are reported as means ± standard deviation (SD). Statistical analyses were performed using GraphPad Prism 9. All statistics and post hoc tests used for multiple comparison correction are stated in the legends.

## Results

### Study design and establishment of the targeted enrichment environment cage

**Figure 1:**
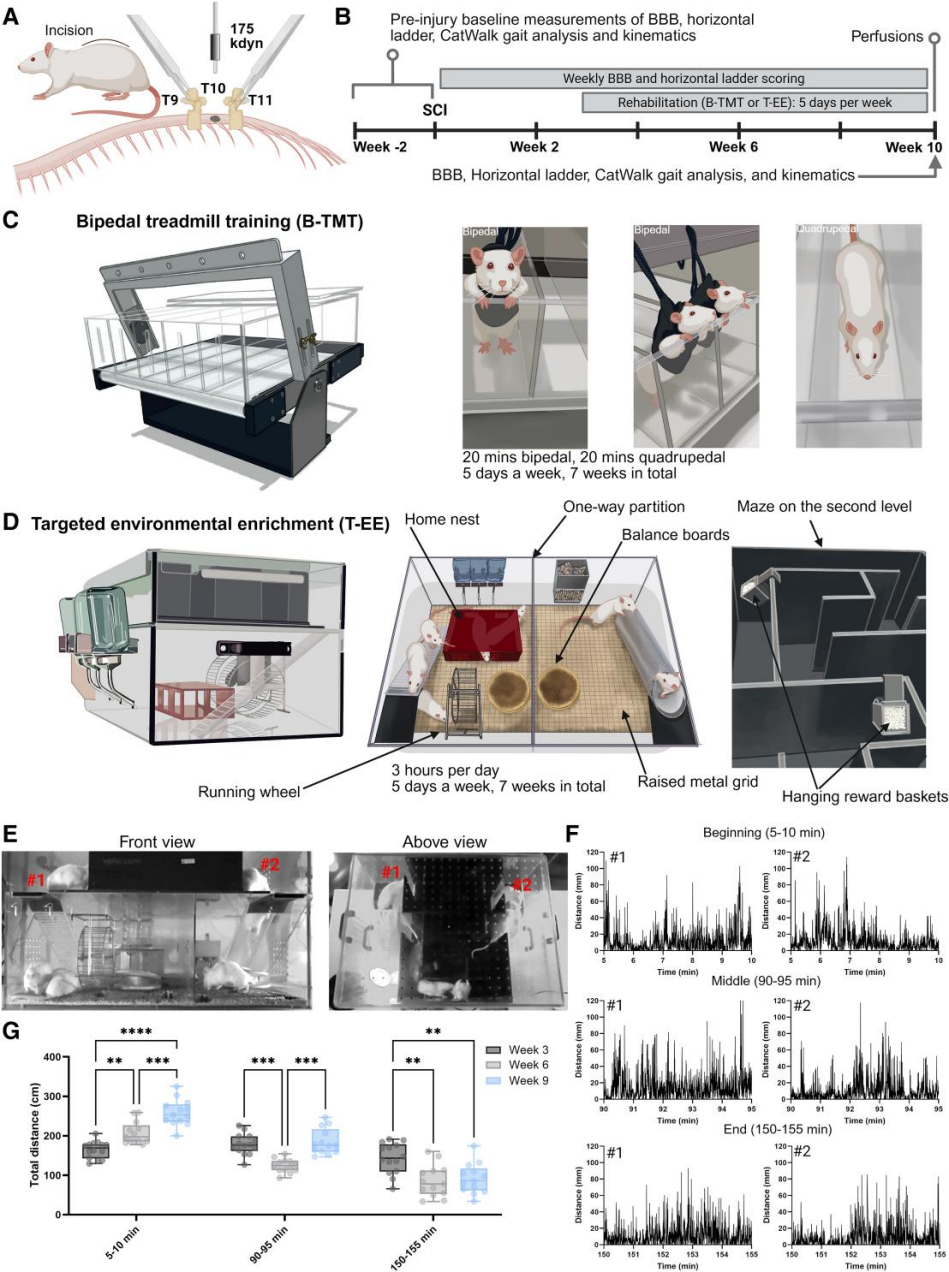
**Study design and setup of the B-TMT and T-EE rehabilitation paradigms.** (**A**) Scheme depicting the location of the moderate T10 175 kdyn contusive injury. (**B**) Scheme of the study design displaying the timing of injury, rehabilitation, and behavioural tests. Created in BioRender. Griffin, J. (2025) https://BioRender.com/ohiqpnk. (**C**) Representative illustrations of the bipedal training paradigm. (**D**) Representative illustrations of the T-EE cage and the task-specific components within it. (**E**) An example of the front and above views recording that were made from week 3, 6 and 9 for three hours each. These recordings were synchronized and calibrated for distance and the movement of individual rats was tracked. (**F**) Examples of two rats tracked between the two synchronized views are marked by the numbers. Movement tracking was quantified within defined time windows of 5–10 min, 90–95 min, and 150–155 min, representing the beginning, middle and end of the 3-h recording sessions. This was then repeated over the weeks 3, 6, and 9 post-injuries. Examples of these traces, represented as distance (mm within 0.2 s), from two rats within the groups are shown. (**G**) Box plots of the group analysis for each time window and week of the recordings. Each dot represents an individual animal. *P < 0.05, **P < 0.01, ***P < 0.001 by repeated measure two-way ANOVA followed by Tukey’s post-hoc tests. (Time of the recording: *F*_1.7, 22.6_ = 109, *P* < 0.0001; Week: *F*_1.7, 21_ = 15, *P* < 0.0002; Time of the recording × Week: *F*_3.3, 38_ = 22, *P* < 0.0001). Week 3 *n* = 14, week 6 *n* = 13, week 9 *n* = 13.

To investigate rehabilitation and epoB efficacy after SCI, we utilized a moderate 175 kdyn thoracic contusion injury (Fig. 1A). Rats received either epoB or vehicle injections on days 1 and 15 post-injury followed by B-TMT or T-EE beginning 3 through to 10 weeks post-injury (Fig. 1B). B-TMT involved partially weight-supported rats completing bipedal stepping on a custom-modified treadmill for 20 min, followed by 20 min of quadrupedal running (Fig. 1C).^9^

The T-EE cage consisted of a two-story design, with the ground floor separated into two compartments by a one-way partition: one side containing food pellets and the other contained water bottles. This encouraged moving and exploring throughout the cage (Fig. 1D). A weekly-rearranged maze was placed on the upper floor to maintain cognitive stimulation. The floor of the ground level and ramp to the second level was covered in a raised metal grid (1 × 1 cm) and balance boards were placed between the one-way partition doors. Rungs were removed in an irregular pattern to the running wheel and baskets of sugar pellets were suspended in the maze at various positions each day. Altogether this provided the task-specific training activities of grid walking, stand training, wheel running, balancing, and grasping—together with social stimulation.

Video recordings of rats in the T-EE cage were captured weekly from three angles—front, above, and side—over a 3-h period (Supplementary Videos 3–6). The front and above views were synchronized and calibrated for distance, enabling accurate tracking of individual rats within the cage (Fig. 1E). We analysed the movement of individual animals during three specific 5-min intervals (5–10 min, 90–95 min, and 150–155 min) across weeks 3, 6, and 9 of the study (Fig. 1F). From the start of the rehabilitation period (week 3), the total distance travelled by the rats during the first analysis time point (5–10 min) increased over time, with distances in week 9 nearly doubling those recorded in week 3 (*P* < 0.0001; Fig. 1G). Activity levels during the mid-session interval (90–95 min) remained relatively consistent across all weeks. However, by the final interval (150–155 min), a decline in activity was observed compared with earlier timepoints. This reduction was more pronounced in weeks 6 and 9 than in week 3 (Fig. 1G). Data from all timepoints followed a normal distribution (Supplementary Fig. 2), and no individual animals exhibited markedly lower activity levels than the group averages (Fig. 1G). These findings suggest that the 3-h sessions appear to be an optimal duration for stimulating activity within the group of animals as a whole within the T-EE cage.

### Targeted environmental enrichment rehabilitation is more effective than bipedal training

**Figure 2:**
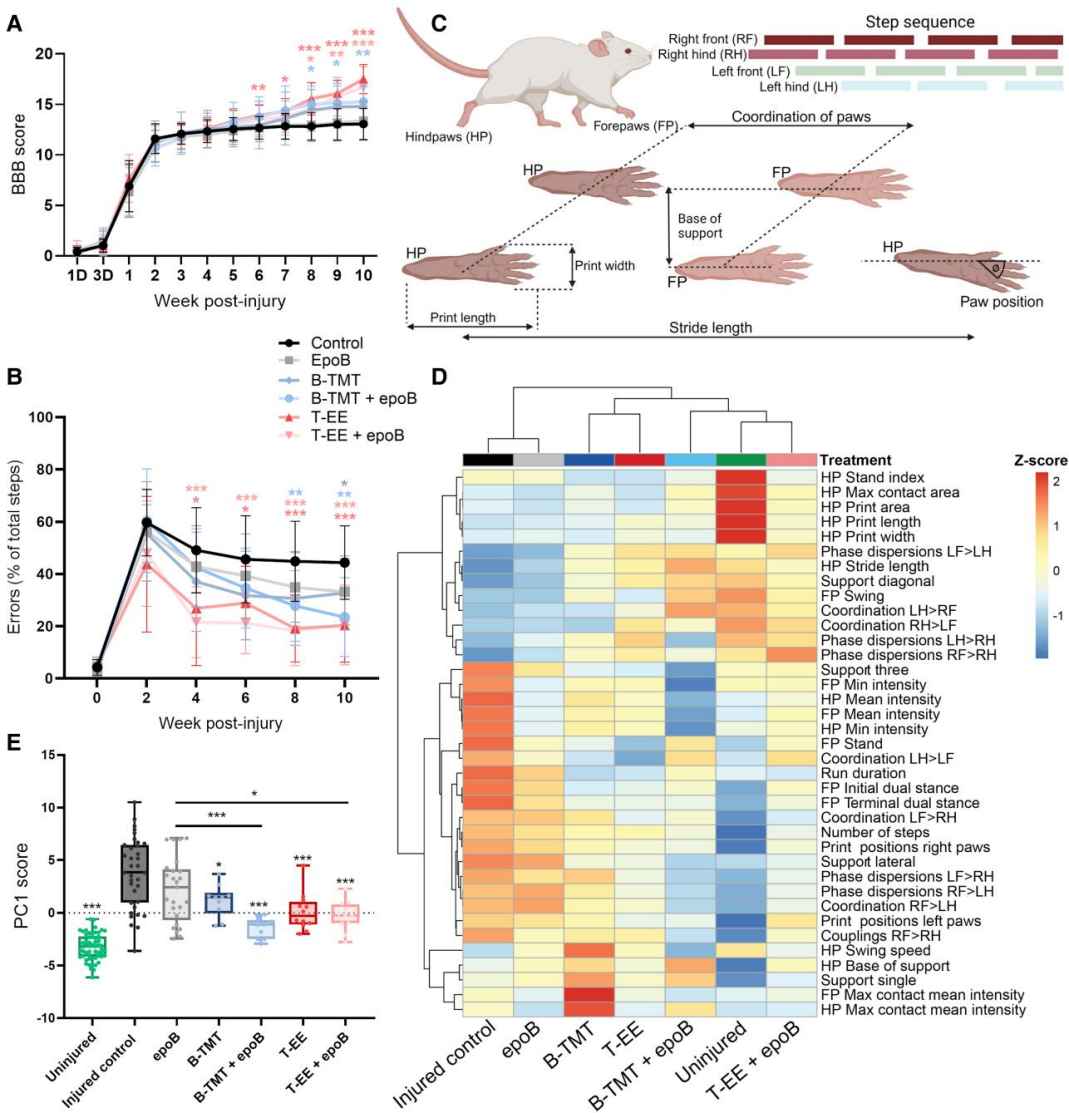
**T-EE is more effective at improving functional behaviour scores compared with B-TMT.** (**A**) BBB scores for the duration of the study. In the first week, scoring was done on days 1 and 3 post-injury: D = day (Time × treatment group: *F*_55, 1023_ = 7.64, *P* < 0.0001; treatment group: *F*_5, 93_ = 2.72, *P* = 0.024). (**B**) Horizontal error ladder scores measured throughout the study. Plotted data for **A** and **B** represent the mean ± SD. *P < 0.05, **P < 0.01, *** *P* < 0.001 by repeated measure two-way ANOVA followed by Tukey’s *post-hoc* tests. (Time × treatment group: *F*_25, 470_ = 4.09, *P* < 0.0001; treatment group: *F*_5,94_ = 6.76, *P* < 0.0001). The colour of the asterisks corresponds to the experimental group being compared with the injured control. Injured control *n* = 29, epoB *n* = 25, B-TMT *n* = 10, B-TMT + epoB *n* = 11, T-EE *n* = 12, T-EE + epoB *n* = 12. Experimental unit is the individual animals. (**C**) A schematic visualization example of some of the parameters measured in the CatWalk gait analysis system. Created in BioRender. Griffin, J. (2025) https://BioRender.com/26uj89q. (**D**) Hierarchical heatmap clustering of the PCA dataset and correlation of parameters between treatment groups. FP, forepaws; HP, hindpaws; RF, right front; LF, left front; RH, right hind; LH, left hind. (**E**) Box plots of the PC1 scores for each animal and statistical analysis of two-way ANOVA with Tukey *post-hoc* where *P < 0.05, **P < 0.01, ***P < 0.001 (*F*_6, 154_ = 40.93, *P* < 0.0001); asterisks without the bars compare to the injured control group. For **D** and **E**, uninjured *n* = 56, injured control *n* = 35, epoB *n* = 25, B-TMT *n* = 10 B-TMT + epoB *n* = 11, T-EE *n* = 12, T-EE + epoB *n* = 12. Experimental unit is the individual animals.

Measurements of force and displacement from the impactor validated that injuries were of equal severity (Supplementary Fig. 3, *P* > 0.05, one-way ANOVA). Injured animals spontaneously recovered BBB scores to reach a final score of 13.0 ± 1.5, and epoB did not improve BBB scores beyond this (Fig. 2A). B-TMT also did not improve BBB scores, reaching a mean score of 14.8 ± 2.0 (*P* = 0.1806). However, there was an improvement when B-TMT was combined with epoB (15.3 ± 1.8; *P* = 0.0088). In contrast, animals exposed to T-EE displayed final mean improvements of 17.5 ± 1.4 (*P* < 0.0001, compared with control) for T-EE alone and 16.7 ± 2.0 (*P* = 0.0006, compared with control) when combined with epoB. Furthermore, T-EE led to significantly greater improvements in BBB scores compared with B-TMT (*P* = 0.024; Fig. 2A; Supplementary Table 3).

Control injured animals made 56% errors on average per total steps in the horizontal ladder test 1 week after the injury, which spontaneously recovered to a final score of 44.4% ± 14.0% (Fig. 2B). Error ladder scoring revealed improvements attributed to epoB treatment 10 weeks post-injury (33.2% ± 12.7%; *P* = 0.0384, compared with control). B-TMT alone did not lead to a reduction in errors (32.7% ± 14.2%; *P* = 0.2392, compared with control), but, when combined with epoB, it led to a significant improvement (23.5% ± 15.0%; *P* = 0.0104). Comparatively, T-EE alone resulted in robust improvements to ladder scores (20.5% ± 14.0%; *P* = 0.0008) that did not appear to be potentiated when combined with epoB (20.5% ± 15.24%; *P* = 0.002 when comparing to control), which may reflect a possible ceiling effect. As such, unlike what was observed for the BBB scores, T-EE also did not improve error ladder scores beyond those achieved with B-TMT (*P* = 0.317; Fig. 2B; Supplementary Table 4).

For a comprehensive analysis of gait and paw function we performed CatWalk gait analysis which analysed 37 parameters including step sequence, paw coordination, print size, paw position and stride length (Fig. 2C; Supplementary Table 2). Values from each parameter and each animal were analysed by principal component analysis (PCA) and hierarchal heatmap clustering. All treatment groups improved various parameters to varying degrees, whilst animals exposed to either form of rehabilitation began to align more closely with uninjured animals in the majority of parameters (Fig. 2D; Supplementary Fig. 4). Notably, the combination of T-EE with epoB resulted in the closest clustering with uninjured animals (Fig. 2D). PC1 alone described one-third of the variance of the data set (Supplementary Fig. 5A) and due to our selection criteria of CatWalk parameters, almost all parameters were highly represented in PC1 (Supplementary Fig. 5B). As such, all treatment groups varied almost exclusively along PC1, indicating that it provides a general evaluation measure of improvements to baseline (Fig. 2E). In contrast, PCs 2 and 3 captured systematic, treatment-specific or interindividual differences (Supplementary Fig. 5B). Uninjured animals scored lowest on PC1 when compared with all other groups, whilst the injured control animals scored highest on PC1. Compared with control injury, all groups improved PC1 scores except for epoB. The greatest improvements, however, were attributed to animals that were exposed to either form of rehabilitation in combination with epoB, which were the most similar to the uninjured group. EpoB improved parameters relating to paw statistics, while both B-TMT and T-EE most effectively improved parameters relating to coordination and limb support (Fig. 2D; Supplementary Fig. 4). When PCs 1–3 are plotted together, there is a notable regression of variability towards the uninjured animals, with T-EE showing the closest alignment (Supplementary Fig. 5C).

**Figure 3.**
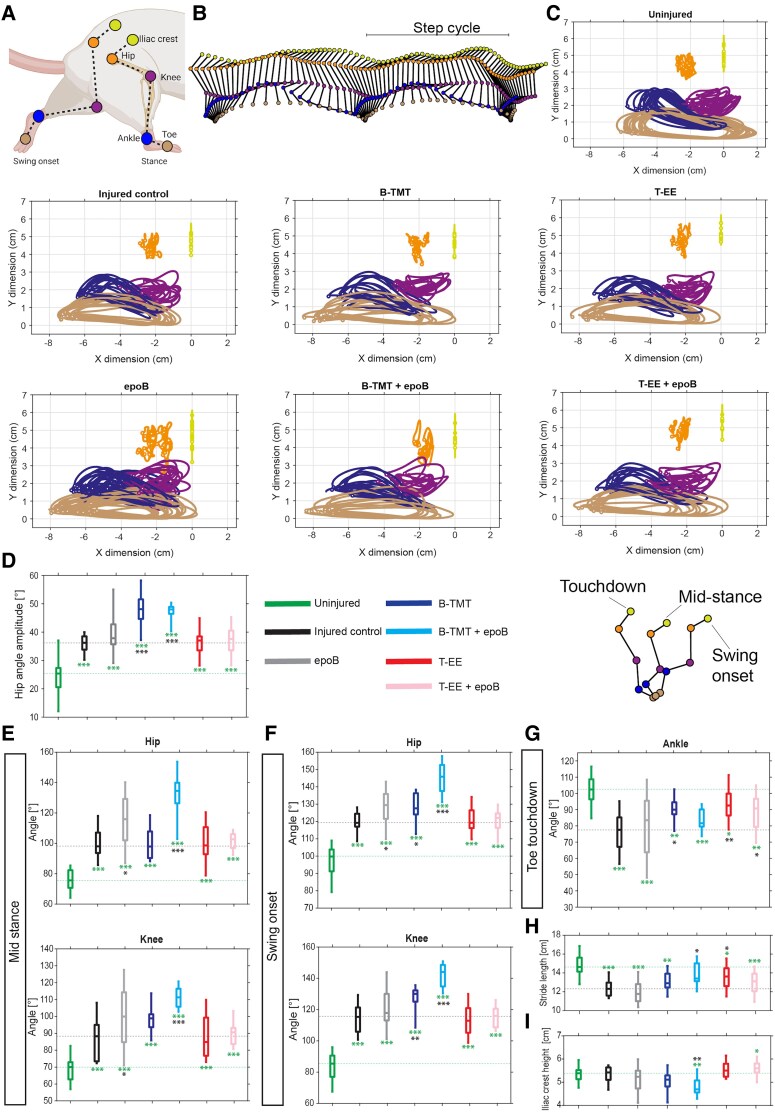
**Hindlimb kinematics during overground walking.** (**A**) Schematic illustration of the five measured marker positions on the hindlimbs; iliac crest, hip, knee, ankle, and toe. Created in BioRender. Griffin, J. (2025) https://BioRender.com/t6uy7qi. (**B**) An example of a walking trial for a right hind leg (uninjured animal). Marker colours correspond to those in **C**. Complete step cycles have been defined as the movement from one lift-off event to the next. (**C**) Stationary step cycles of the tracked joints in relation to the position of the iliac crest. Each average trial per animal and group is shown. (**D**) The total amplitude of the hip for the normalized joint angle time-courses (see Supplementary Fig. 5 for the full joint angle time-courses). Analyses were further subjugated to touchdown, mid-stance, and swing onset. Box plots of the joint angles of the hip and knee measured at (**E**) mid-stance, (**F**) swing onset and (**G**) touchdown. Box plots of the measured (**H**) step cycle stride-lengths and (**I**) iliac crest height during swing onset. *P < 0.05, **P < 0.01, ***P < 0.001 by Wilcoxon rank-sum test; black asterisks indicate comparisons with the injured control group, whereas green asterisks indicate comparisons with the uninjured group. Green dashed lines show the median of the uninjured group, whilst grey dashed lines show the median of the injured control group. Uninjured *n* = 17, injured control *n* = 12, epoB *n* = 25, B-TMT *n* = 10, B-TMT + epoB *n* = 11, T-EE *n* = 12, T-EE + epoB *n* = 12. Experimental unit is the individual animals.

Lastly, we assessed hindlimb kinematics during overground stepping by tracking the iliac crest, hip, knee, ankle, and toe. The data were segmented into step cycles (Fig. 3A and B) and further normalized to joint angle time courses (Supplementary Fig. 6A) and stationary step cycles (Fig. 3C). After SCI, a posterior shift in the position of the joints in relation to the iliac crest was noted, along with decreased joint angles across all joints (Fig. 3C; Supplementary Fig. 6A). In general, injured animals recovered kinematic profiles to a high degree. However, bipedal step-trained animals displayed larger hip amplitudes and increased joint angles of the hip and knee during mid-stance and swing onset (Fig. 3D–F). Similar changes to joint angles of the hip and knee could be partially observed for animals treated with epoB. This is in opposition to the profile of uninjured animals, which may reflect postural changes imposed through bipedal stepping or may be compensatory in nature. These changes were not seen in the T-EE, and T-EE + epoB groups, which could reflect resilience to such treatment-induced alternative kinematic profiles. Both B-TMT and T-EE improved ankle angle during hindpaw touchdown, resulting in increased stride length (consistent with the gait analysis; Fig. 3G and H). Lastly, T-EE resulted in higher iliac crest heights during swing onset (Fig. 3I), mid-stance and touchdown (Supplementary Fig. 6B), which may indicate greater hindlimb weight-bearing capacity and strength.

In summary, our findings demonstrate that while all treatment groups exhibited varying degrees of functional recovery following SCI, the most pronounced improvements were observed in animals exposed to T-EE, both alone and in combination with epoB.

## Discussion

To achieve meaningful translational success in SCI research, it is essential to combine the most effective therapeutic interventions with optimized, reproducible rehabilitation protocols.^13^ Historically, rehabilitation strategies for thoracic-level SCI have focused almost exclusively on quadrupedal or B-TMT.^3,4^ While these paradigms have demonstrated some efficacy, they have several limitations: restricted rehabilitation duration, variability between handlers, increased animal stress, and a narrow focus on specific movement patterns. To address these limitations, we developed a standardizable rehabilitation approach based on EE that incorporates task-specific elements—we decided to term this type of rehabilitation as T-EE. In comparison to ‘general EE’, which typically solely includes social housing and nesting materials,^14^ T-EE is designed to actively promote functional recovery through deliberate inclusion of rehabilitative tasks. This approach minimizes user intervention, extends the duration and diversity of rehabilitative movements, and may enhance both welfare and experimental reproducibility.^15^ EE, as described by the Five Freedoms Domains of Animal Welfare,^16^ supports animals’ ability to express natural behaviours and is widely recognized for improving welfare. While EE has been widely recognized for addressing welfare concerns,^16,17^ some studies have reported adverse effects, such as increased aggression,^18^ though this was not noted in our study.

In our study, we found that T-EE improved functional outcomes compared with B-TMT, producing a ceiling effect in recovery metrics at the moderate injury we used. This suggests that T-EE may be more appropriately targeted toward rehabilitation following severe contusion injuries. Although previous studies have reported mixed outcomes with EE following SCI,^19,20^ those incorporating task-specific components (i.e. T-EE) have shown promising results.^21-25^ For example, Starkey *et al*.^21^ demonstrated that a self-motivated T-EE paradigm led to significant improvements in locomotor function, ladder-climbing, and swimming performance after severe thoracic SCI.^21^ Similar findings have been reported across multiple studies, with no evidence of behavioural decline.^22-26^ To fully evaluate the relative benefits of T-EE across preclinical models, a comprehensive meta-analysis will be essential. Such an analysis would help clarify its efficacy compared with other rehabilitation approaches and refine its optimal target injury severity.

The mechanisms underlying EE—and particularly T-EE—remain complex and not fully understood. Evidence suggests that EE promotes synaptic plasticity via increased BDNF expression, modulates immune responses to favour regeneration, and stimulates angiogenesis to enhance nutrient delivery and tissue repair.^6^ EE may also facilitate motor and sensory circuit reorganization.^6,27-29^ However, an overly neurocentric perspective may be reductive. The benefits of EE-based rehabilitation may also stem from peripheral mechanisms, such as improved muscle strength through sustained activity, enhanced cardiovascular health, and better mood regulation.^30-32^ It may also be possible that the concept of ‘use it or lose it’ comes into context here. Experimental evidence supports the principle that limb disuse leads to functional decline: for example, rats with one forelimb immobilized post-SCI or stroke exhibited significant deficits in that limb.^33^ Similar findings have been replicated after SCI,^34^ reinforcing the importance of activity-dependent plasticity. In enriched environments, it may be that exposing the animals to a wider range of movements is beneficial towards improving a wider array of functional outcomes and that actions that are not attended to become attenuated. However, further investigation is needed to disentangle these mechanisms.

In conclusion, integrating T-EE with therapeutic interventions offers a promising and practical strategy for enhancing functional recovery after SCI. Its reproducibility, minimal handling requirements, and broad rehabilitative scope make it a strong candidate for both preclinical research and future clinical translation.

## Supplementary Material

fcaf385_Supplementary_Data

## Data Availability

All data represented in each figure from this article, including the supplementary figures, has been uploaded to Mendeley Data Repository and is publicly available under the DOI: 10.17632/pbp3hp8dxz.1. This can be discovered through searching for the article title within the Mendeley Data Repository search bar. Due to the size of the data, video recordings from the kinematics, CatWalk gait analysis and horizontal error ladder behavioural analyses are archived with the DZNE repository and available upon request.
