# Supplementary material for: Targeted environmental enrichment is more effective than bipedal treadmill training after thoracic spinal cord injury
Source: Brain Commun. 2025 Oct 6;7(5):fcaf385. doi: 10.1093/braincomms/fcaf385 (PMC12529096; doi:10.1093/braincomms/fcaf385)
Supplement: fcaf385_Supplementary_Data [file fcaf385_supplementary_data.zip › Supplementary_material.pdf]

Supplementary Material

Targeted environmental enrichment is more effective than bipedal treadmill training after thoracic spinal cord injury

Jarred Griffin, Till Bockemühl, Blanca Randel, Stefanie Gröschl, Panagiotis Papaioannou, Ansgar Büschges, and Frank Bradke.

Extended methods

Animals, surgical procedures and epothilone treatment

Experiments were performed per Animal Welfare Act and the North Rhine-Westphalia State Environment Agency guidelines and in accordance with the ARRIVE guidelines. Adult female Sprague Dawley rats (Janvier Labs) weighing approximately 250 g were housed in a reverse light cycle. The animals were administered analgesics (Meloxicam, 1 mg/kg, subcutaneously (s.c.); Boehringer Ingelheim, Germany) and antibiotics (Enrofloxacin, 5 mg/kg, s.c.; Elanco, Germany) and then anaesthetised with isoflurane. A laminectomy was performed at spinal level T10 and impacted with a force of 175 kdyn using the Infinite Horizon Impactor (U.S.A). The overlying musculature and skin were sutured and 0.6 g/kg glucose was administered s.c. The animals were then transferred to a heated cage to recover for at least an hour before being returned to a clean home cage. Animals were administered antibiotics (Enrofloxacin 5 mg/kg, s.c.) analgesics (Meloxicam 1 mg/kg, and Buprenorphine 0.05 mg/kg; s.c. C.P-Pharma, Germany) for three days following the surgery. Animals were randomly allocated to intervention groups and received blinded s.c. injections of epoB (0.75 mg/kg; Selleck Chemicals, USA, #S1364), or vehicle (50% DMSO, Carl Roth, Germany, #200-664-3) on days 1 and 15 post-injury.

Bipedal treadmill training rehabilitation

Bipedal treadmill training was conducted three weeks after the injury. A custom-adapted five-lane rodent treadmill (Pan Lab, Spain, #LE8710RTS) was used whereby animals were supported upright in harnesses and positioned so that their hind paws touched the surface of the treadmill, as previously described.[1,2] This apparatus and harness allowed the animals to remain upright whilst performing near full weight-bearing movements with their hindlimbs. Training, beginning one week prior to the injury, involved a three-day-long habituation period, during which rats were acclimated to the treadmill and suspension harnesses, followed by three days of guided hindlimb walking for 30 minutes per session. By three weeks post-SCI, all the animals recovered at least weight-supported plantar stepping and were therefore able to be trained effectively for bipedal stepping. The rats completed bipedal training at a speed of 20 to 30 cm/s for 20 min a day, 5 days a week, for 7 weeks in total. On average, this totalled 400 meters of running on the hindlimbs per day. To illustrate the training process, we have added two supplementary videos: one from the pre-training phase and another from the post-injury training phase (**Supplementary Videos 1 and 2**). These highlight both the method used to train the

animals to walk bipedally and their ability to perform B-TMT at speeds ranging from 20 to 30 cm/s. Immediately following bipedal training, the rats underwent 20 min of quadrupedal exercise training at a speed of 30 to 40 cm/s.

Targeted environmental enrichment rehabilitation

The Marlau environmental enrichment cage (ViewPoint, France, #MARR1) served as the basis of the T-EE cage which offers complexity, activity, novelty and animal well-being. The cage features a two-story design, including a ground floor with two compartments separated by a one-way door: one containing food pellets and the other water bottles, and an upper floor housing a replaceable maze. Imposing such a path to procure food or water ensures all animals have equal access to the different features of enrichment, and cognitive stimulation is maintained by weekly changes in maze configuration. From there, modifications were made to cover the floor with a raised grid to encourage intentional placement of the hindlimbs and grasping of the paws; an irregular rung running wheel was added; balance boards were placed between the partition doors to stimulate body balancing; reward baskets loaded with sugar pellets were hung in the maze and changed position every day to encourage standing and exploratory behaviour. A maximum of 14 rats were placed together in the cage. Each week, complete recordings of the total 3 hours were made from three viewpoints of the cage to confirm and assess activity within the cage (**Supplementary Videos 3-6**).

Activity analysis

For quantitative analysis of the recordings from the T-EE cage, tracking of individual animals within the cage was conducted at weeks 3, 6, and 9 post-injuries. From each three-hour-long weekly recording, three 5-min-long segments were selected for analysis: 5-10 min, 90-95 min, and 150-155 min. Distance measurements were conducted using Fiji and the MTrackJ plugin. Videos were calibrated to distance and MTrackJ was used to manually track each rat's x/y coordinates in mm at every frame of the recording, with the time interval between frames set at 0.2 s. The real distances between successive coordinates were calculated and summed to determine the total distance travelled.

BBB scoring and horizontal ladder test

One week prior to injury, all animals were habituated and trained to perform each behavioural task for 30 min daily over three days. Basso, Beattie and Bresnahan (BBB) scoring was carried out by two blinded observers in a circular open field for 5 min, as previously described (Basso et al., 1995). For the horizontal ladder test, a 100 cm-long ladder with irregularly spaced rungs was used. Each animal completed three runs of the ladder while being recorded using a GoPro Hero5. The footage was analysed frame-by-frame by a blinded observer to capture footfalls and total steps. Steps were considered an error if the paw slipped from the rung, with both partial and full slips included. The average number of errors was recorded over the three runs and expressed as a percentage of the total steps.

CatWalk gait analysis

The CatWalk XT Gait analysis system was used to quantitate parameters of gait and locomotion in the rats one week prior to their injury and at ten weeks post-injury. Only runs with a

maximum run variation of less than 60% and completion within five seconds were included. Four successful runs per animal were recorded. Gait parameters are represented as an average of the four runs per animal, per time point. Parameters that displayed a fold change of 15% between uninjured and injured animals with a $P < 0.01$ were included in the study (**Supplementary Fig. 1, Supplementary Tables 1 and 2**). This data set was centred, and each column normalised to a standard deviation of 1 before calculating PCA. We extracted the explained variance for each principal component (PC) and the coefficients for the first three PCs. Following this, we processed this PCA dataset by collapsing columns with similar annotations to the means and performed heatmap hierarchical clustering to identify correlations between groups.

2D kinematic recordings and analysis

Data acquisition was conducted as previously described (Griffin et al., 2023). Markings were made on the following landmarks of the hindlimbs: iliac crest, greater trochanter (hip), lateral malleolus (ankle), lateral epicondyle (knee) and the metatarsophalangeal joint of the fifth toe (**Fig. 3A**). Videos of overground walking were recorded at a frame rate of 193 frames per second. The markers were tracked using TSE Motion V9.2.2 pattern recognition software generating x and y coordinates for all markers on both body sides over the time course of the runs. In addition, for each side view, we manually added annotation that we used as a reference line for the ground.

To segment the continuous motion data into individual steps, we used the absolute speed of the toe marker in global coordinates as a proxy to detect onsets of stance (touchdown events) and swing phases (lift-off events), respectively. When the leg touches down, the toe contacts the ground and therefore becomes largely stationary; this was defined as onset of stance. Conversely, when the leg lifts off, the toe starts moving again with respect to the ground. Thus, times at which the speed of the toe marker dropped below an empirically determined threshold (16.7% of the median of the maximum speeds during the trial) were identified as stance onset and those at which it exceeded this threshold were identified as swing onset. A complete step, consisting of a swing phase and the subsequent stance phase, was then defined as the movement of the leg between two swing onset events. All further analyses were based on complete individual steps.

Steps differed in duration, so in order to average the steps for each animal, we normalized the time course of marker positions for each step (swing onset to swing onset) to 100 equidistant data points. These normalized steps were then averaged, resulting in a dataset comprising 100 points per marker for the average step. Based on these normalized average steps, we calculated average marker positions throughout steps, average joint angle time courses and their respective amplitudes, joint angles at swing onset, mid-stance, and stance onset, as well as the average iliac crest height during trials and the average stride length.

Average marker positions throughout average steps were calculated as their global x and y-positions from which the respective x-positions of the iliac crest marker were subtracted, i.e. the forward component of walking. Therefore, the resulting data depict a stationary

representation of leg movements. Joint angles were defined as the inner angle either between a proximal and the next distal segment of the leg or, in the case of the hip, the angle between a constant horizontal vector and the first segment. Amplitudes were defined as the absolute difference between the minimum and maximum value for a particular joint angle. Stride length was defined as the absolute global distance between the toe position at swing onset and its position at the subsequent touchdown. Iliac crest height was defined as the average distance between the iliac crest marker and the ground reference line for a complete trial.

Data exclusion and statistical analyses

Data in the present study includes reanalysed data from our previous publication [1]. Exclusion criteria were set prior to the initiation of the study and included: a combined hindlimb BBB score greater than five at day three after surgery; failure to improve beyond a combined score of nine by the end of the three-week period; or euthanasia due to surgical complications. Statistical analyses were performed using GraphPad Prism 9. To assess whether data sets followed a normal distribution, a D'Agostino & Pearson normality test with $\alpha = 0.05$ was applied to check for Gaussian distribution. Data sets passed this test and as such, parametric one-way and two-way ANOVA statistical analyses were applied. The exception to this was the statistical analysis for the kinematic data which utilised the Wilcoxon rank-sum test. All statistics and post hoc tests for multiple comparison correction are stated in the text where appropriate. For all analyses: $*P < 0.05$, $**P < 0.001$, $***P < 0.0001$.

Supplementary Tables:

Supplementary Table 1. Raw list of CatWalk parameters with fold-change of about 0.15 and *P* < 0.01. FP = forepaws, HP = hindpaws, RF = right front, LF = left front, RH = right hind, LH = left hind.

Parameter	Fold-change value
PrintPositions_RightPaws_Mean_(cm)	-0.88344
PrintPositions_LeftPaws_Mean_(cm)	-0.8038
Support_Lateral_(%)	-0.63857
PhaseDispersions_LF->RH_CStat_Mean	-0.63269
PhaseDispersions_RF->LH_CStat_Mean	-0.63235
Couplings_RF->LH_CStat_Mean	-0.61141
PhaseDispersions_RF->LH_Mean	-0.60765
Couplings_LF->RH_CStat_Mean	-0.6014
PhaseDispersions_LF->RH_Mean	-0.59592
Support_Single_(%)	-0.55556
FP_InitialDualStance_(s)_Mean	-0.55312
FP_TerminalDualStance_(s)_Mean	-0.54985
HP_StandIndex_Mean	-0.30291
HP_MinIntensity_Mean	-0.30187
Support_Three_(%)	-0.27319
OtherStatistics_NumberOfSteps	-0.26465
FP_MaxContactMeanIntensity_Mean	-0.25162
FP_MeanIntensity_Mean	-0.246

HP_MeanIntensity_Mean	-0.245
HP_MaxContactMeanIntensity_Mean	-0.23552
FP_MeanIntensityOfThe15MostIntensePixels_Mean	-0.20302
FP_MinIntensity_Mean	-0.20216
BOS_HindPaws_Mean_(cm)	-0.19656
Couplings_LH->LF_Mean	-0.18718
Couplings_RF->RH_CStat_Mean	-0.18123
FP_Stand_(s)_Mean	-0.17533
OtherStatistics_Duration_Mean	-0.17019
HP_StrideLength_(cm)_Mean	0.158233
HP_SwingSpeed_(cm/s)_Mean	0.163271
PhaseDispersions_LH->RH_Mean	0.164698
FP_Swing_(s)_Mean	0.197175
Couplings_RH->LF_CStat_Mean	0.227104
Couplings_LH->RF_CStat_Mean	0.24864
PhaseDispersions_LF->LH_Mean	0.305563
Couplings_LH->RF_Mean	0.306325
Couplings_RH->LF_Mean	0.368125
PhaseDispersions_RF->RH_Mean	0.449945
HP_PrintLength_(cm)_Mean	0.451168
Couplings_RF->LH_Mean	0.460218
HP_MaxContactArea_(cm²)_Mean	0.465676
HP_PrintWidth_(cm)_Mean	0.524552
Couplings_LF->RH_Mean	0.54547
HP_PrintArea_(cm²)_Mean	0.62786
Support_Diagonal_(%)	0.836931

Supplementary Table 2. Refined list of parameters analysed in the CatWalk Gait analysis, and their classifications. FP = forepaws, HP = hindpaws, RF = right front, LF = left front, RH = right hind, LH = left hind.

	Gait parameter	Stance
FP stand	Paw statistics	Stand
FP Max contact mean intensity	Paw statistics	Intensity
FP Min intensity	Paw statistics	Intensity
FP Mean intensity	Paw statistics	Intensity
FP Swing	General	Speed
FP Initial dual stance	Gait	Stance
FP Terminal dual stance	Gait	Stance
HP Stand index	Gait	Stance
HP Max contact area	Paw statistics	Area
HP Max contact mean intensity	Paw statistics	Intensity
HP Print length	Paw statistics	Length
HP Print width	Paw statistics	Width
HP Print area	Paw statistics	Area

HP Min intensity	Paw statistics	Intensity
HP Mean intensity	Paw statistics	Intensity
HP Swing speed	General	Speed
HP Stride length	General	Stepping
HP Base of support	Support	Base of support
Run duration	General	Speed
Number of steps	General	Stepping
Print positions right paws	Paw statistics	Position
Print positions left paws	Paw statistics	Position
Phase dispersions RF>LH	Phase dispersion	Diagonal
Phase dispersions LF>RH	Phase dispersion	Diagonal
Phase dispersions LH->RH	Phase dispersion	Contralateral
Phase dispersions RF->RH	Phase dispersion	Contralateral
Phase dispersions LF>LH	Phase dispersion	Ipsilateral
Coordination RF>LH	Coupling	Diagonal
Coordination LF>RH	Coupling	Diagonal
Coordination LH>RF	Coupling	Diagonal
Coordination RH>LF	Coupling	Diagonal
Coordination RF>RH	Coupling	Ipsilateral
Coordination LH->LF	Coupling	Ipsilateral
Support single	Support	Single
Support diagonal	Support	Diagonal
Support lateral	Support	Lateral
Support three	Support	Three

Supplementary Table 3. Statistical analyses of the BBB locomotor rating score at 10 weeks post-injury. A two-way ANOVA with Tukey's multiple comparison *post-hoc* test was done to compare the differences between individual experimental groups.

Comparison	Mean diff	95% CI of diff	Summary	P Value
Injured control vs. epoB	-0.3283	-1.756 to 1.100	ns	0.9829
Injured control vs. T-EE	-4.448	-6.026 to 2.870	****	<0.0001
Injured control vs. T-EE + epoB	-3.615	-5.736 to 1.494	***	0.0006
Injured control vs. B-TMT	-1.748	-4.027 to 0.5301	ns	0.1806
Injured control vs. B-TMT + epoB	-2.221	-4.153 to 0.2886	*	0.0196
epoB vs. T-EE	-4.12	-5.855 to -2.385	****	<0.0001
epoB vs. T-EE + epoB	-3.287	-5.508 to -1.065	**	0.0018
epoB vs. B-TMT	-1.42	-3.780 to 0.9396	ns	0.4172
epoB vs. B-TMT + epoB	-1.893	-3.938 to 0.1527	ns	0.0803
T-EE vs. T-EE + epoB	0.8333	-1.453 to 3.120	ns	0.8557
T-EE vs. B-TMT	2.7	0.2832 to 5.117	*	0.0241
T-EE vs. B-TMT + epoB	2.227	0.1058 to 4.349	*	0.0362
T-EE + epoB vs. B-TMT	1.867	-0.8497 to 4.583	ns	0.2971
T-EE + epoB vs. B-TMT + epoB	1.394	-1.095 to 3.883	ns	0.5148
B-TMT vs. B-TMT + epoB	-0.4727	-3.070 to 2.124	ns	0.9913

Supplementary Table 4. Statistical analyses of the horizontal error ladder score at 10 weeks post-injury. A two-way ANOVA with Tukey's multiple comparison *post-hoc* test was done to assess the differences between individual experimental groups.

Comparison	Mean diff	95% CI of diff	Summary	P Value
Injured control vs. epoB	11.16	0.3818 to 21.94	*	0.0384
Injured control vs. T-EE	24.06	8.968 to 39.15	***	0.0008
Injured control vs. T-EE + epoB	23.88	7.727 to 40.03	**	0.002
Injured control vs. B-TMT	11.6	-4.342 to 27.54	ns	0.2392
Injured control vs. B-TMT + epoB	20.88	4.102 to 37.66	*	0.0104
EpoB vs. T-EE	12.9	-2.121 to 27.93	ns	0.1192
EpoB vs. T-EE + epoB	12.72	-3.369 to 28.80	ns	0.1732
EpoB vs. B-TMT	0.442	-15.44 to 16.32	ns	>0.9999
EpoB vs. B-TMT+ epoB	9.723	-7.001 to 26.45	ns	0.4545
T-EE vs. T-EE + epoB	-0.1856	-18.82 to 18.45	ns	>0.9999
T-EE vs. B-TMT	-12.46	-30.91 to 5.990	ns	0.3179
T-EE vs. B-TMT + epoB	-3.18	-22.28 to 15.92	ns	0.9946
T-EE + epoB vs. B-TMT	-12.28	-31.48 to 6.930	ns	0.3752
T-EE + epoB vs. B-TMT + epoB	-2.994	-22.81 to 16.82	ns	0.9966
B-TMT vs. B-TMT + epoB	9.281	-10.36 to 28.93	ns	0.6768

Supplementary Figures:

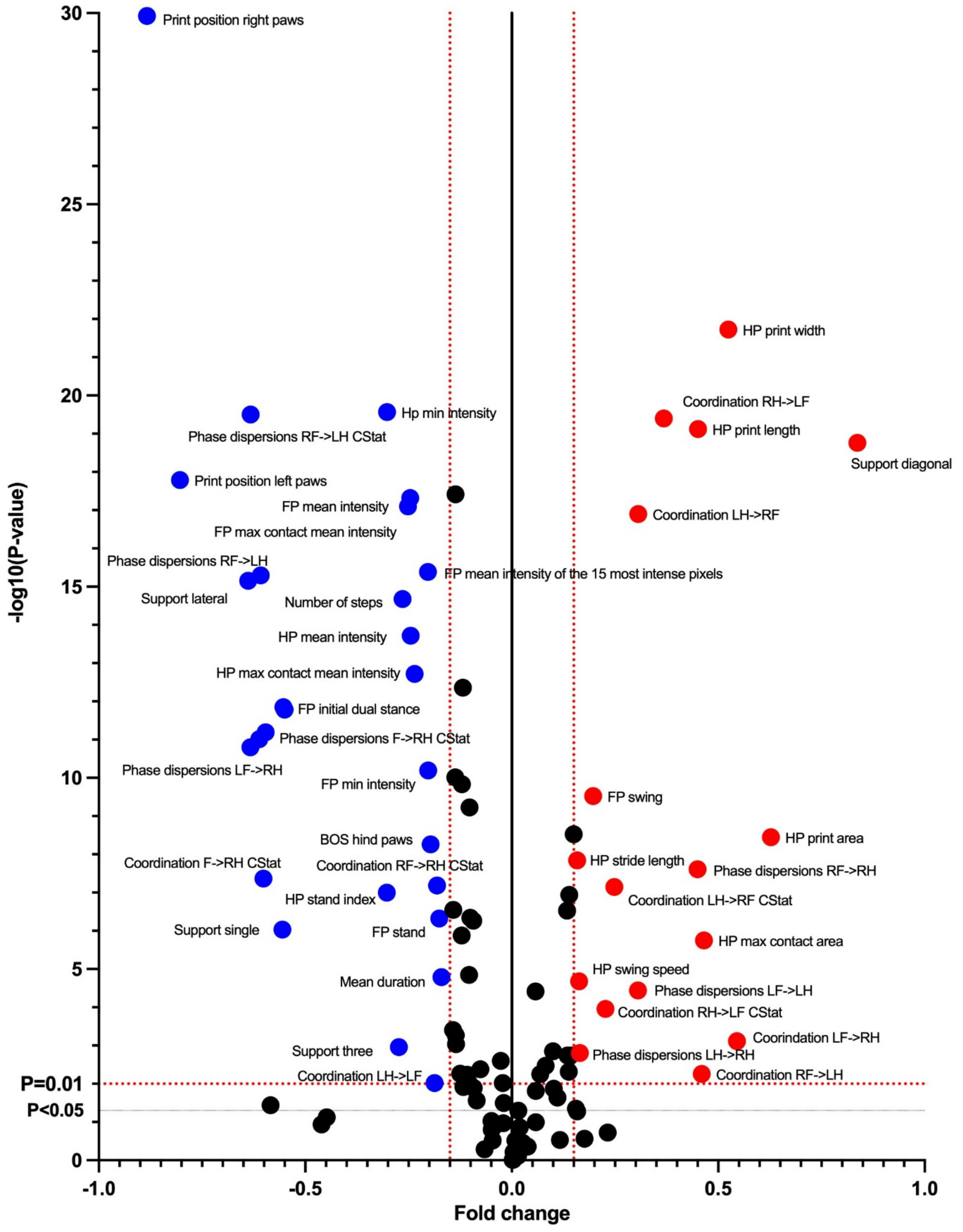

Supplementary Figure 1. Volcano plot of CatWalk gait analysis parameters. A non-biased selection process of gait parameters was performed by plotting a volcano plot of parameters that displayed a fold change of ± 15% from uninjured to injured animals with a *P* value of less than 0.01. This resulted in a total of 44 parameters, presented in supplementary table 1. This was then refined to the total of 37 parameters shown in supplementary table 2.

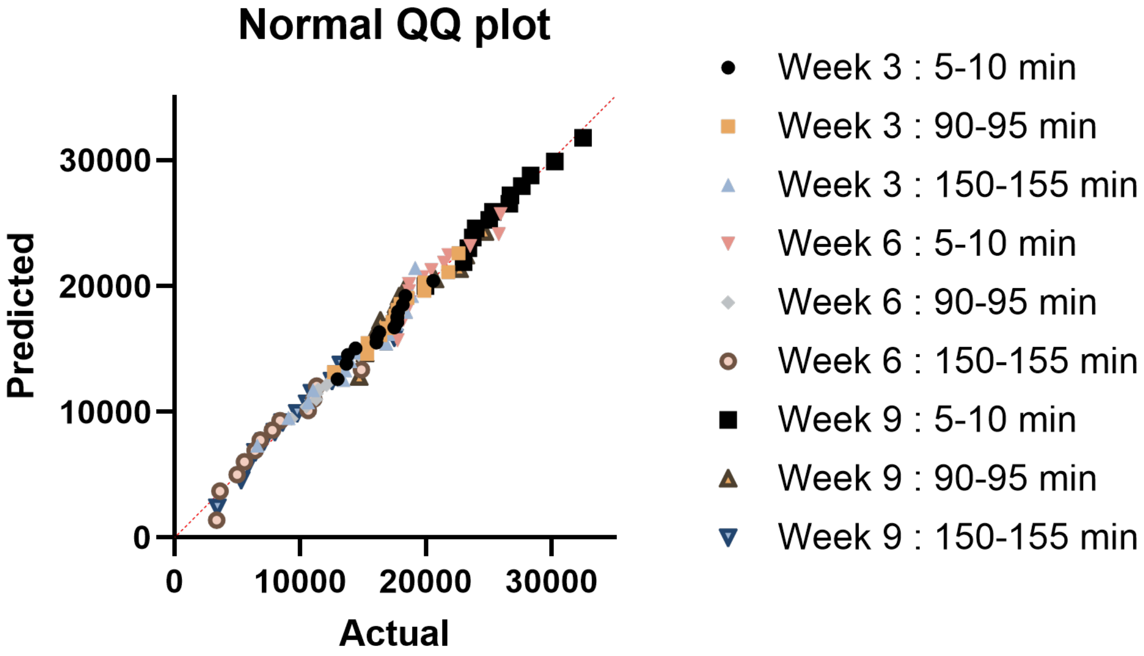

Supplementary Figure 2. QQ plot of the data from all weeks and time periods. Normal distribution of the data was tested through D'Agostino-Pearson omnibus normality test and represented as a QQ plot (week 3 5-10 min $P = 0.9270$; week 3 90-95 min $P = 0.9008$; week 3 150-155 min $P = 0.5736$; week 6 5-10 min $P = 0.2887$; week 6 90-95 min $P = 0.9890$; week 6 150-155 min $P = 0.5910$; week 9 5-10 min $P = 0.7549$; week 9 90-95 min $P = 0.3793$; week 9 150-155 min $P = 0.4597$).

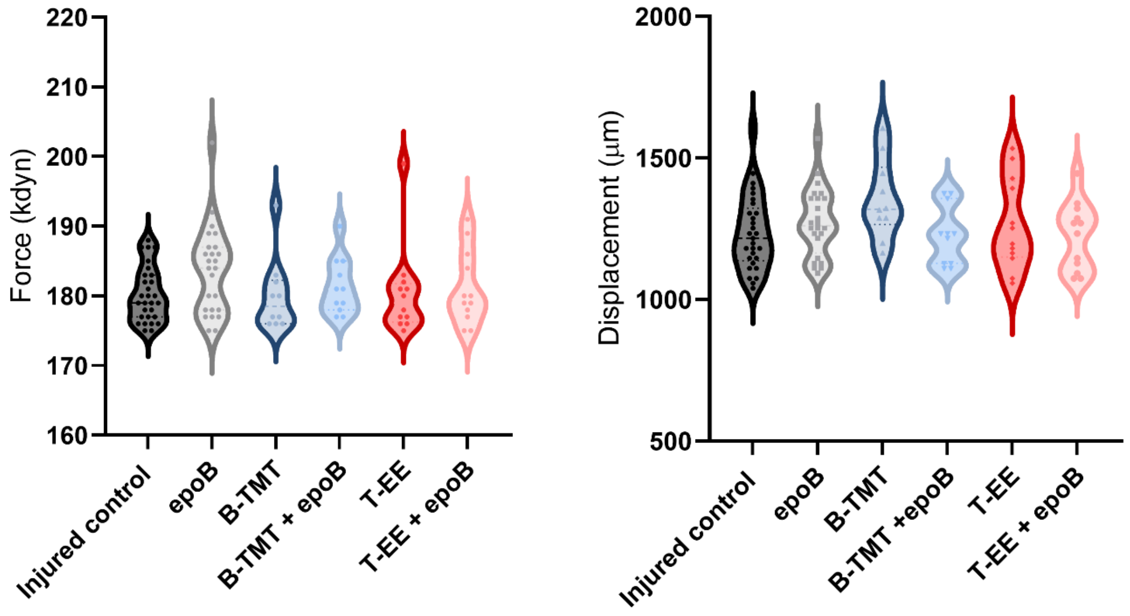

Supplementary Figure 3. Force and displacement values for each experimental group. Actual force and displacement measurements from each impaction were plotted and analysed using one-way ANOVA with Bonferroni *post-hoc*. Injured control $n = 29$, epoB $n = 25$, B-TMT $n = 10$, B-TMT + epoB $n = 11$, T-EE $n = 12$, T-EE + epoB $n = 12$.

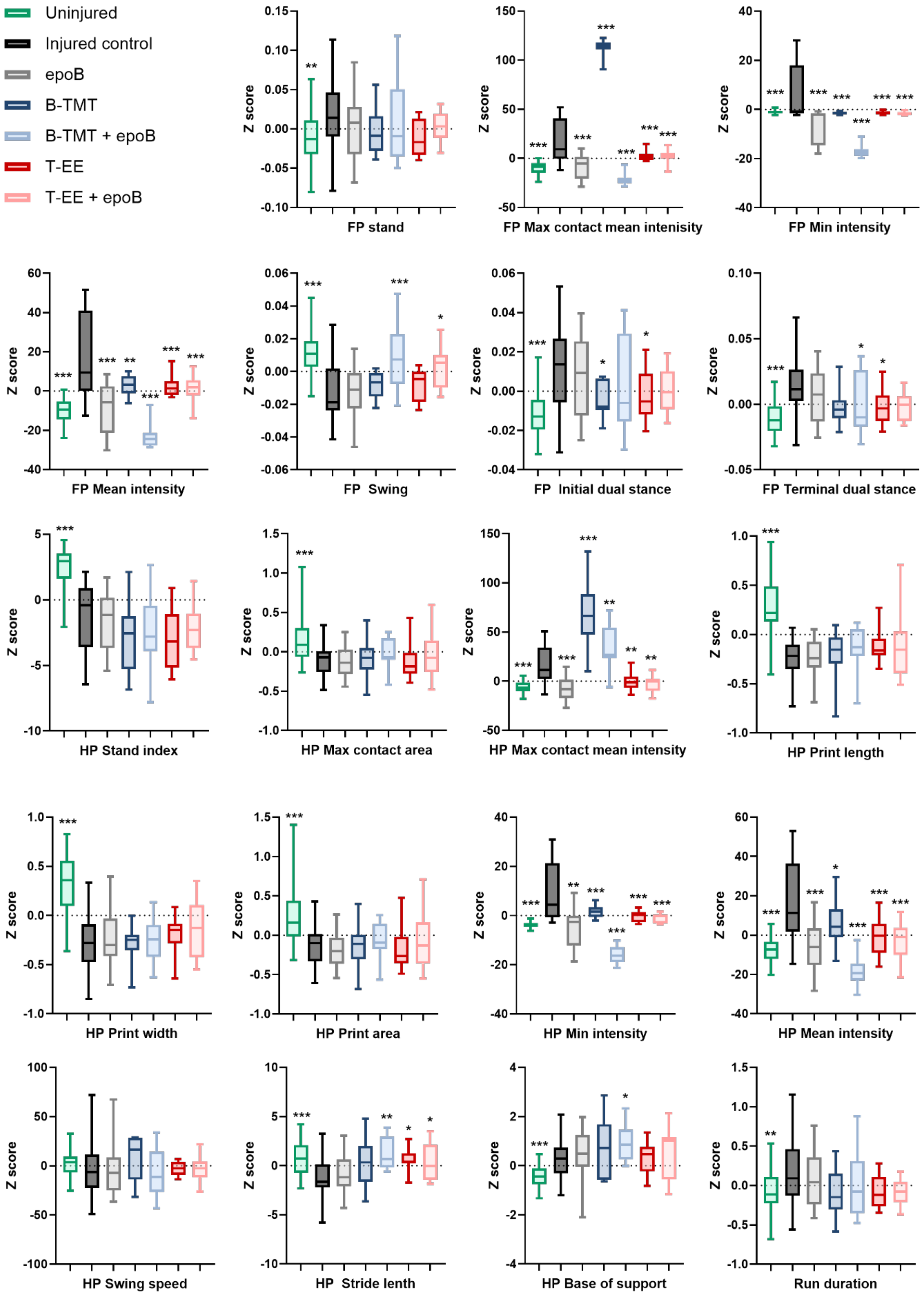
Uninjured
Injured control
epoB
B-TMT
B-TMT + epoB
T-EE
T-EE + epoB
Z score
FP stand
FP Max contact mean intenisity
FP Min intensity
FP Mean intensity
FP Swing
FP Initial dual stance
FP Terminal dual stance
HP Stand index
HP Max contact area
HP Max contact mean intensity
HP Print length
HP Print width
HP Print area
HP Min intensity
HP Mean intensity
HP Swing speed
HP Stride lenth
HP Base of support
Run duration

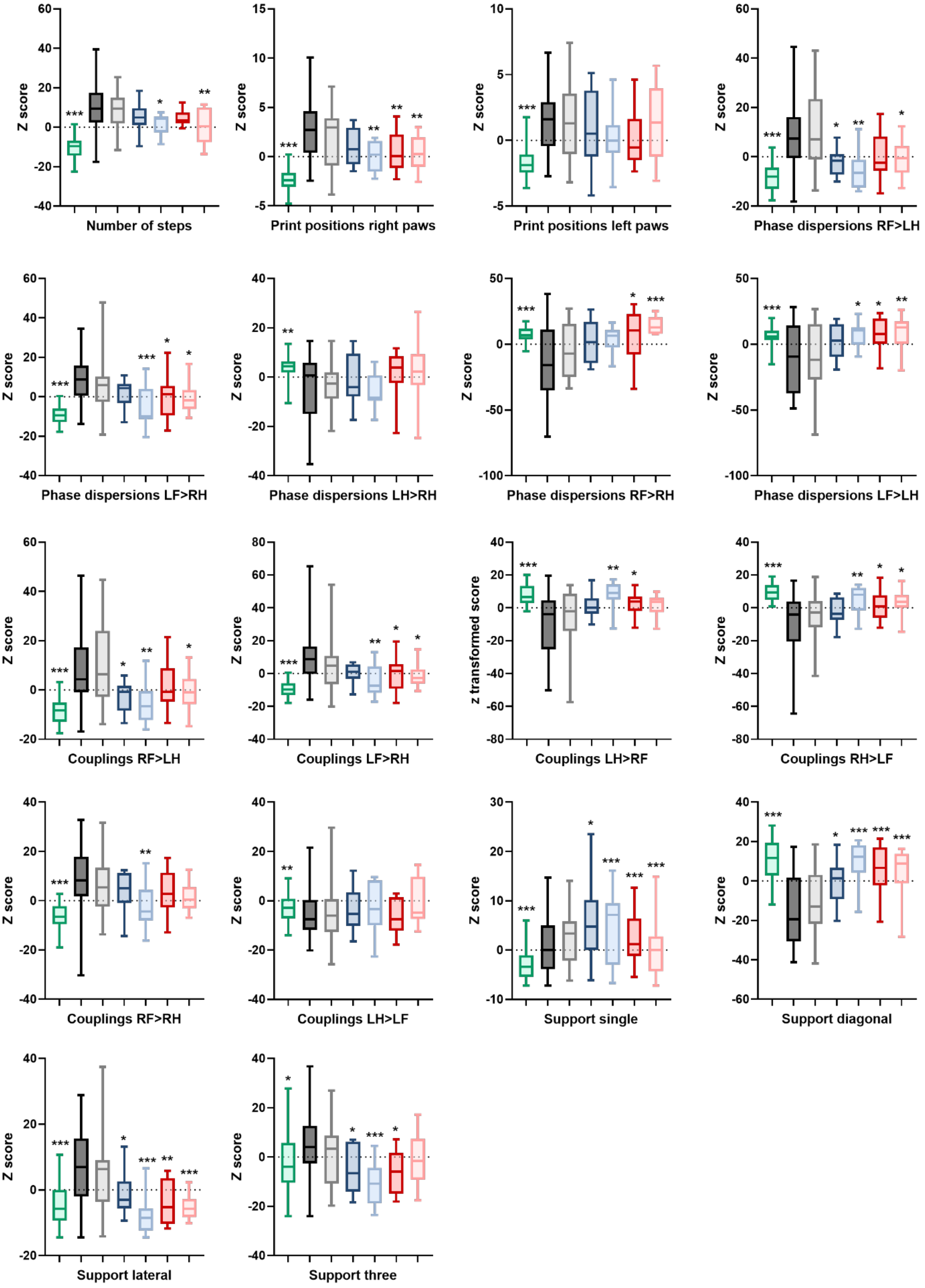

Supplementary Figure 4. Individually plotted parameters of the CatWalk gait analysis dataset. Individual graphs were plotted for each parameter used in the CatWalk data analysis (Fig. 2) and

presented in the order that they appear in Supplementary Table 2. *$P < 0.05$, **$P < 0.01$, ***$P < 0.001$, ****$P < 0.0001$ by one-way ANOVA and Dunnett's *post-hoc* where the comparisons are to the injured control group. Uninjured $n = 56$, Injured control $n = 35$, epoB $n = 25$, B-TMT $n = 10$, B-TMT + epoB $n = 11$, T-EE $n = 12$, T-EE + epoB $n = 12$.

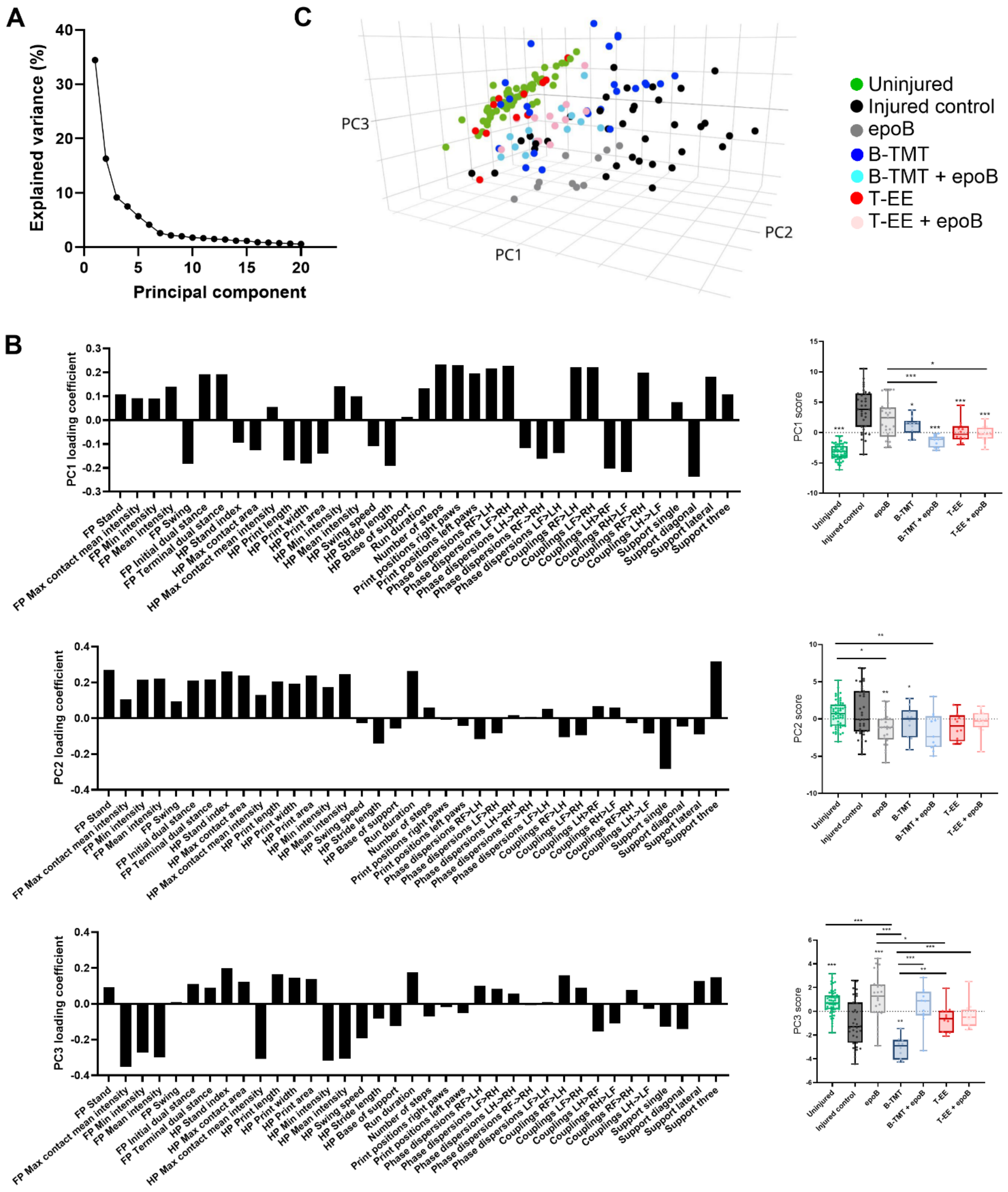

Supplementary Figure 5. Principal component variance and loading coefficients. (A) Plot of explained variance for each principal component in the PCA. **(B)** Loading coefficient values for each parameter for principal components 1, 2 and 3, along with their associated PC scores. *$P < 0.05$, **$P <$

0.01, ***$P < 0.001$, ****$P < 0.0001$ by one-way ANOVA and Tukey's *post-hoc.* **(C)** Three-dimensional plot of PC's 1, 2 and 3 together. Uninjured $n = 56$, Injured control $n = 35$, epoB $n = 25$, B-TMT $n = 10$, B-TMT + epoB $n = 11$, T-EE $n = 12$, T-EE + epoB $n = 12$.

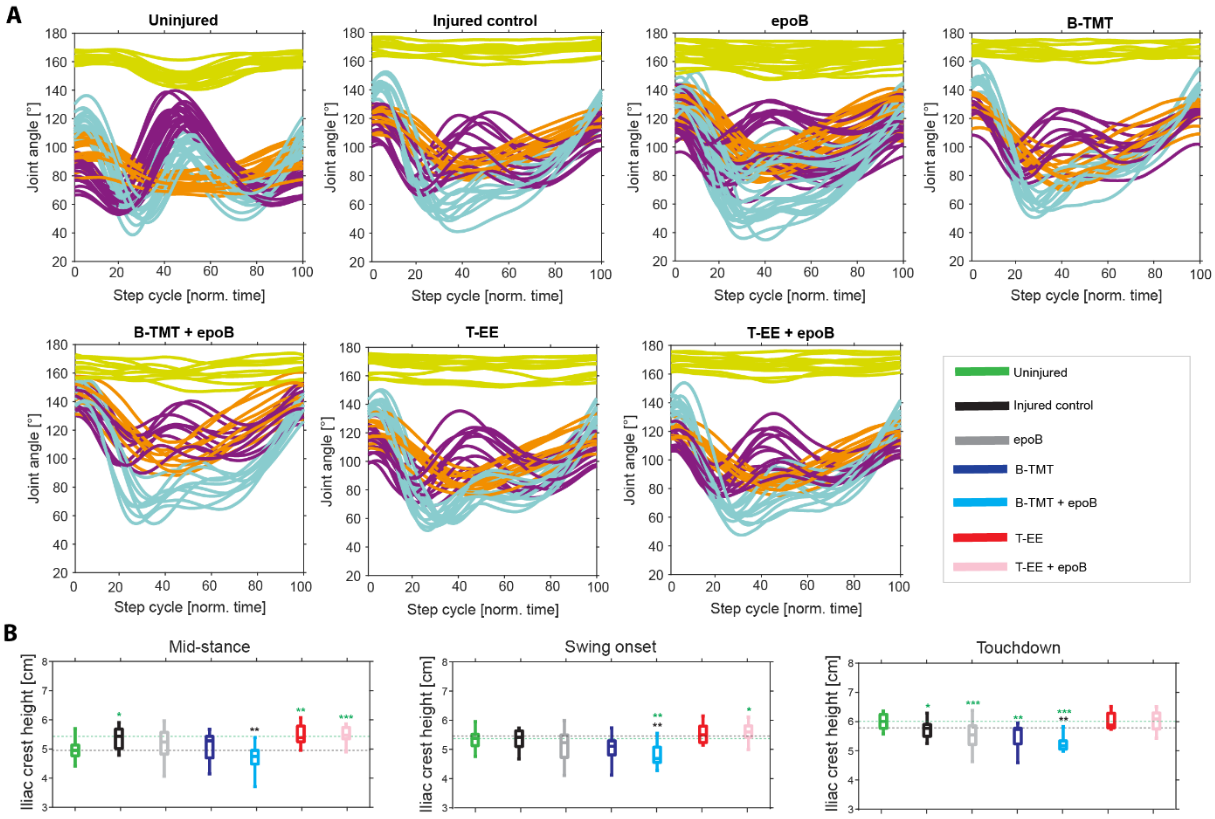

Supplementary Figure 6. Additional kinematics data. **(A)** Individual average joint angle time-courses of iliac crest (yellow), hip (orange), knee (purple), and ankle (blue) from normalised step cycles. **(B)** Box plots of iliac crest height at mid-stance, swing onset and touchdown. *$P < 0.05$, **$P < 0.01$, ***$P < 0.001$ by Wilcoxon rank-sum test; black asterisks show comparisons to the injured control, whereas green asterisks show comparisons to the uninjured group. Uninjured $n = 18$, injured control $n = 12$, epoB $n = 25$, B-TMT $n = 10$, B-TMT + epoB $n = 11$, T-EE $n = 12$, T-EE + epoB $n = 12$.

<u>Supplementary Videos:</u>

Supplementary Video 1. Demonstration of the bipedal treadmill training procedure prior to spinal cord injury. Before spinal cord injury, rats underwent a structured training protocol to learn bipedal treadmill walking. The training began with individual sessions, where each rat was first supported by allowing it to grasp a finger of the trainer with its forelimbs. This was followed by training the animal to hold onto the edge of the treadmill enclosure. Once the rats became accustomed to upright posture and movement, they were fitted into support jackets and gradually transitioned to independent bipedal locomotion on the treadmill.

Supplementary Video 2. Bipedal treadmill training as a rehabilitation procedure following spinal cord injury. Three weeks after spinal cord injury, rats began bipedal treadmill training as part of their rehabilitation. The process starts with individual re-training to re-establish the bipedal stepping pattern. Once the animals regained sufficient coordination, up to five rats were trained simultaneously. If an animal fell or turned sideways during training, it was gently repositioned to maintain proper orientation. Throughout the study, treadmill speeds ranged between 20 to 30 cm/s.

Supplementary Video 3. Demonstration of activity in the targeted enrichment environment rehabilitation over a 5 min period. Video recordings were made during the three hours of rehabilitation in the targeted environmental enrichment cage every week for the duration of the study. A 5-minute example of a recording period is shown. Three cameras were used to record the top, side and front views of the cage.

Supplementary Video 4. Demonstration of activity in the targeted enrichment environment rehabilitation at week 3 after the injury. A high-frame-rate version of the recording from the front view of the targeted environmental enrichment cage during the third week of the study.

Supplementary Video 5. Demonstration of activity in the targeted enrichment environment rehabilitation at week 6 after the injury. A high-frame-rate version of the recording from the front view of the targeted environmental enrichment cage during the sixth week of the study.

Supplementary Video 6. Demonstration of activity in the targeted enrichment environment rehabilitation at week 9 after the injury. A high-frame-rate version of the recording from the front view of the targeted environmental enrichment cage during the ninth week of the study.

References

1. Griffin JM, Hingorani Jai Prakash S, Bockemuhl T, et al. Rehabilitation enhances epothilone-induced locomotor recovery after spinal cord injury. *Brain Commun*. 2023;5(1):fcad005. doi:10.1093/braincomms/fcad005

2. Griffin JM, Fackelmeier B, Clemett CA, et al. Astrocyte-selective AAV-ADAMTS4 gene therapy combined with hindlimb rehabilitation promotes functional recovery after spinal cord injury. *Exp Neurol*. May 2020;327:113232. doi:10.1016/j.expneurol.2020.113232
